# Optimizing an Electronic Health Record System Used to Help Health Care Professionals Comply With a Standardized Care Pathway for Heart Failure During the Transition From Hospital To Chronic Care: Qualitative Semistructured Interview Study

**DOI:** 10.2196/63665

**Published:** 2025-04-15

**Authors:** Marta Font, Nadia Davoody

**Affiliations:** 1 Department of Learning, Informatics, Management and Ethics Health Informatics Centre Karolinska Institutet Stockholm Sweden

**Keywords:** care pathway, heart failure, electronic health record, sociotechnical system, health care professional

## Abstract

**Background:**

In Spain, the prevalence of heart failure is twice the European average, partly due to inadequate patient management. To address this issue, a standardized care model, the Care Model for Patients with Heart Failure (Modelos Asistenciales de Atención al Paciente con Insuficiencia Cardíaca), was developed. This model emphasizes the importance of sequential visits from hospital discharge until the patient transitions to chronic care to prevent rehospitalization. The standardized care pathway has been implemented in certain areas of the Andalusia Health Service. However, there is uncertainty about whether the region’s electronic health record system, Diraya, can effectively support this model. If not properly integrated, it could lead to data inaccuracies and noncompliance with the standardized care pathway.

**Objective:**

This study aimed to explore how to improve Diraya to better support health care professionals in adhering to the transition standardized care model for patients with heart failure as they move from hospital care to chronic care.

**Methods:**

In total, 16 semistructured interviews were conducted with nurses and physicians from both hospital and primary care settings. Thematic analysis was used to analyze the data and recommendations for improvements that were developed based on the findings. These recommendations were further supported by existing literature and validated through additional interviews.

**Results:**

In total, 65 codes, 23 subthemes, and 8 themes were identified. The main themes included optimizing medical data management for enhanced clinical workflow, agreement on standardization and enhancement of the discharge report, enhancing clinical decision support through updated guidelines and automated tools, optimizing interoperability as a solution for better management of patients with heart failure, and encouraging communication based on digital tools and personal connection. In total, 15 improvements were proposed, such as standardizing technology across Andalusia Health Service facilities and offering targeted training programs. These measures aim to enhance interoperability, streamline communication between different health care settings, and reduce the administrative burden for health care professionals.

**Conclusions:**

Diraya currently does not adequately support the transition standardized care model, placing a significant administrative burden on health care professionals, often with ethically concerning implications. To ensure effective implementation of the standardized care model, major updates are necessary for Diraya’s clinical information management, system functionality, and organizational structure within the Andalusia Health Service.

## Introduction

### Heart Failure

Chronic diseases, such as heart failure (HF), pose a significant public health burden, threatening not only population health but also social and economic stability. Consequently, both public health strategies and health care delivery systems must undergo transformative changes [1]. Diagnosing HF requires identifying cardiac dysfunction, often linked to various risk factors [2]. Early detection, timely intervention, and continuous monitoring—from primary care to hospitalization—are crucial in the recovery process [3].

HF was first identified as an emerging epidemic in 1997 [4], and it remains a major health concern today, with a prevalence of approximately 2% among adults in Europe and North America [2,5-7]. In Spain, however, the prevalence is nearly double, with an annual incidence rate of 2.78 per 1000 patients [8].

Managing HF is particularly challenging due to its chronic nature, severe symptoms, and significant impact on patients’ quality of life. Furthermore, HF imposes a substantial economic burden, primarily driven by frequent hospitalizations and long-term health care needs [9]. To address these challenges, population health management and integrated care pathways have emerged as effective strategies. These approaches aim to improve health care systems’ responsiveness, better meet patient needs and preferences, and enhance efficiency [9]. A key example of such an approach is the standardized care pathway (SCP), which provides structured guidance for the medical treatment of patients with specific clinical conditions, ensuring high-quality care [5]. SCPs offer detailed, standardized treatment plans for patient groups with predictable clinical courses, leading to improved outcomes, more efficient resource use, and enhanced clinical effectiveness—ultimately increasing patient satisfaction [9]. Given its benefits, implementation of an SCP for HF is crucial, as it provides a systematic, evidence-based framework for the comprehensive management and treatment of patients with HF [10].

### Spanish National Health System

#### Overview

The Spanish National Health System (SNHS) encompasses Spain’s public health services [11]. It operates under a decentralized model, where autonomous communities are responsible for managing and delivering health services, while the Ministry of Health establishes general guidelines and coordinates national health policies [12].

Within SNHS, HF is the leading cause of hospitalizations among patients aged >65 years, accounting for 5% of all hospital admissions. It is estimated that HF-related hospitalizations will increase by up to 50% over the next 25 years [3]. The economic burden of HF is substantial, costing approximately €2.5 billion (US $2.7 billion) annually, which represents 3.8% of Spain’s total health care expenditure [13].

To improve HF management, Spain has implemented a comprehensive continuous care program for patients with HF, which focuses on patient and family education, comprehensive assessment, and continuity of care [14]. Despite these efforts, cardiovascular diseases remain the leading cause of hospitalization and mortality in Spain and effectively managing patients with HF continues to be a challenge [15]. To address this issue, Spain developed a standardized care model known as the Care Model for Patients with HF, (Modelos Asistenciales de Atención al Paciente con Insuficiencia Cardíaca [MAIC]), aimed at improving the quality and coordination of HF care across health care settings [16].

#### Spanish Care Model for Patients With HF

The Care Model for Patients with HF project is led by several key medical societies in Spain, including the Spanish Society of Cardiology, the Spanish Society of Internal Medicine, the Spanish Society of Primary Care Physicians, the Spanish Society of Family and Community Medicine, the Spanish Society of Healthcare Executives, and the Spanish Society of Hospital Pharmacy, with Antares Consulting and Boehringer Ingelheim as investors [16]. To address challenges in pathology and optimize patient care, these organizations developed a comprehensive document in 2020, synthesizing current literature and interviews with health care professionals (HCPs). This document includes implementation tools, key considerations for HF care as a SCP, and a flowchart detailing the entire patient journey—from hospitalization to chronic treatment and follow-up (Multimedia Appendix 1 [16]).

**Figure 1:**
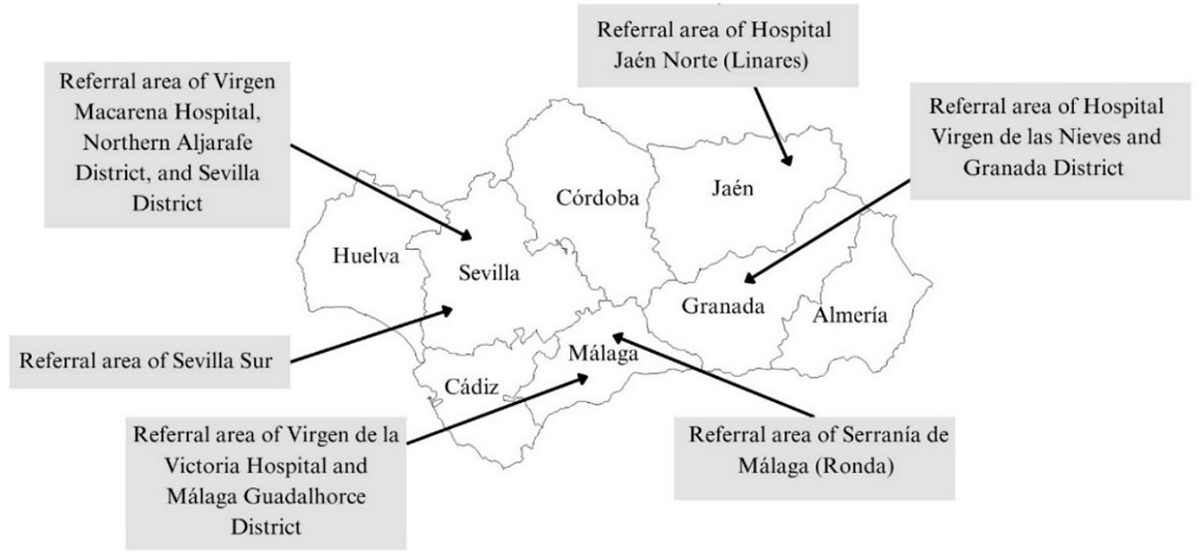
Map of Andalusia highlighting each referral area where the standardized care pathway has been implemented. Courtesy of Antares Consulting Company.

The transition from hospital discharge to outpatient follow-up is a critical phase for ensuring continuity of care, as it significantly impacts patient prognosis, the risk of decompensation, and the likelihood of hospital readmissions. Figure 1 highlights the main transition SCP (TSCP), which focuses on patient discharge from the hospital to primary care.

After hospital discharge, a hospital nurse (HN) and a cardiologist or internal medicine (C/IM) specialist oversee the patient transition. Within the first 48 to 72 hours, a primary care nurse (PCN) contacts the patient by phone to check their condition. Shortly after, the patient has an in-person visit with both a PCN and a primary care physician (PCP) for further assessment. Finally, 2 to 3 weeks later, the C/IM specialist conducts a follow-up visit, ensuring the patient’s transitions to chronic HF care. The standardized care model, MAIC [16]. was implemented during the first semester of 2024 in 5 hospitals and their respective primary care centers in Spain, with 29 additional hospitals in the process of adopting this standardized care model. The implementation follows a 6-8–month timeline during which health care centers are evaluated and monitored for adherence to the care model. The progress tracking covers a year, using process and outcome indicators from health care data to assess improvements.

### Andalusia Health Service

The TSCP is currently in operation across several autonomous communities in Spain, including Andalusia. Located in the southern part of the country, Andalusia is the most populous and the second largest autonomous community. The Andalusia Health Service (AHS) is committed to providing accessible, equitable, and high-quality public health care services to its citizens [17]. The AHS consists of a network of health care services, organized into 15 health areas, which include 5 regional hospitals, 10 specialized hospitals, 19 district hospitals, and 16 high-resolution hospitals [18]. Each health area is structured around a reference hospital and its associated primary care centers, ensuring comprehensive coverage for the population. In terms of HF, approximately 140,000 individuals have been diagnosed with the condition in Andalusia [19].

In December 2023, the AHS initiated the implementation of the SCP in 6 hospitals and their corresponding primary care settings, collectively designated as referral areas, across Andalusia (Figure 1). The goal is to expand this implementation throughout the autonomous community.

The TSCP involves 4 key actors, each playing a crucial role in the process. These actors come from 2 health care settings, hospital and primary care, and represent 2 professions: physicians and nurses. Each participant has distinct responsibilities, performs specific tasks, and receives different training (Table 1). However, their collaboration is crucial to achieving better outcomes for patients with HF.

**Table 1:** Description of the health care professionals’ roles involved in the care of patients with heart failure.

Role	TSCP^a^ role description
Cardiologist or internal medicine specialist	Performs the discharge assessment and a visit after 2-3 wk of the discharge. The main tasks are as follows: manage and assess the discharge report; medication management; communication with primary care, that is, reviewing hospital reports
Hospital nurse	Performs the nurse assessment for the patient discharge. The main task that is performed is to manage and assess the discharge report.
PCN^b^	Does the first evaluation after the discharge, via phone call. Then they perform a face-to-face visit. The main tasks that are performed are as follows: educate, check warning signs, and titrate medication (blood test and writing the report, among other); manage upcoming and follow-up visits in the unit; communicate with the hospital, that is, review hospital reports.
PCP^c^	First medical evaluation after the discharge. This visit must be completed in less than a week. The main tasks are as follows: conduct the initial face-to-face and subsequent follow-up visits after discharge (assessment and medical history among writing the report, among others); manage medication; communicate with the hospital, that is, review hospital reports

^a^TSCP: transition standardized care pathway.

^b^PCN: primary care nurse.

^c^PCP: primary care physician.

### Electronic Health Records for HF Practice

To successfully implement an SCP, electronic support, particularly an electronic health record (EHR) system, is essential [20]. The EHR needs to align with the different steps of the SCP and meet the specific needs of HCPs [21]. Diraya, the EHR system used by the AHS, was implemented in 2005 and has undergone multiple updates to adapt to the evolving demands of the health care sector [22].

Diraya consists of various modules that share information across different health care environments and among professionals. These interconnected components ensure that each piece of data are recorded uniquely and can be accessed when needed [22]. For the care of a patient with HF, Diraya uses the following modules in both hospital and primary care settings [23]: (1) the Primary Care Clinical History module allows for the visualization and editing of data, including personal information, health programs, care processes, medical history, allergies, prescriptions, medication orders, medication, work disabilities, and follow-up records by the family doctors; (2) Hospital Clinical History module in hospitals, excluding emergencies, manages care processes associated with clinical profiles from the perspective of hospital care, generating basic and specialized reports; (3) Hospital Clinical Care handles nursing records related to patient care and organizes them into structures reports; (4) Mailbox manages automated notifications to professionals in primary and hospital care, serving as a key communication channel, for example, when a patient with HF is discharged, their PCP receives a notification; and (5) Single Navigator provides access to all reports, regardless of their origin.

The implementation of EHR systems like Diraya enhances health care services by streamlining medical information management. However, a key challenge remains: does Diraya effectively support HCPs in managing care for patients with HF within the SCP?

### Aim of the Study

In Spain, patients with HF who are discharged without specific measures face a high risk of readmission within the first month. Optimizing Diraya is imperative to improving care for patients with HF and ensuring better adherence to the SCP. Achieving this requires a comprehensive understanding of the needs of HCPs involved in the transition from hospital to primary care. To our knowledge, no studies have explored the optimization of an EHR system for patients with HF transitions based on HCPs’ needs in adhering to an SCP—either within the AHS or in other Spanish regions.

This study aimed to identify ways to improve Diraya to better support HCPs in following the TSCP for patients with HF during the transition from hospital to chronic care. The research focuses on the clinical content, human-computer interaction, technical aspects, and organizational and communication needs in HF care within the EHR and TSCP. In addition, it seeks to provide a list of recommended improvements for Diraya.

## Methods

### Study Design

The study used a qualitative approach to gain an in-depth understanding of HCPs’ needs. Semistructured interviews with open-ended questions were chosen as the data collection method. The sociotechnical system (STS) model [24] served as both the framework and the interview guide for this study. The interviews were analyzed using thematic analysis, combining both deductive and inductive reasoning to thoroughly explore the data. Following the analysis, drafts summarizing the results and highlighting relevant improvements to meet HCPs’ needs were created. These results were then verified and validated with a representative from each health care setting. This validation process adhered to the principles of qualitative research methodology, ensuring the reliability and credibility of the data collected.

### Study Setting and Participants

The study was conducted across 6 health care areas of AHS where the SCP was in the process of being implemented. The areas were as follows: (1) referral area of Virgen Macarena Hospital, Northern Aljarafe District, and Sevilla District; (2) referral area of Hospital Jaén Norte; (3) referral area of South Sevilla; (4) referral area of Virgen de la Victoria Hospital and Málaga Guadalhorce District; (5) referral area of Serranía de Málaga; and (6) referral area of Hospital Virgen de las Nieves and Granada District.

To ensure the participation of relevant HCPs involved in the TSCP across both health care settings and during the discharge transition, 4 key roles were included: PCPs, PCNs, HNs, and C/IM specialists. Participants were selected using purposive sampling [25], and to expand the participant pool, snowball sampling was also applied [26]. Sampling continued until data saturation was achieved.

Initial contact with potential participants was made via email, with follow-up phone calls or WhatsApp (Meta Platforms) messages when necessary. During these interactions, a concise overview of the study, including its aims and objectives, was provided. Interested participants received an informed consent document, which they were required to sign and return before the interview. Recruitment was facilitated through Antares Consulting [27].

The inclusion criteria required participants to be actively engaged in the care of patient with HF, employed within the AHS, and working in either hospitals or primary care settings as nurses or physicians. The study excluded participants with <3 years of experience and those currently on sick leave. The limited number of participants and the focus on specific areas in Andalusia may limit the generalizability of our study. Future research should include a larger and more diverse participant base. This limitation is discussed further in the Discussion section.

### Data Collection

To address the research question, data were collected through interviews guided by the STS model. In addition, the identified needs and corresponding improvements were verified through follow-up interviews.

#### Interviews

The interviews were conducted individually, using a semistructured format, with open-ended questions. The process was carried out in 2 phases: the first phase explored the needs of HCPs, while the second phase verified the proposed improvements. For the second phase, 2 participants with different roles were selected from each health care setting.

All interviews were conducted via Microsoft Teams, recorded, and transcribed verbatim using the platform’s transcription feature. They were conducted in Spanish, the native language of both the participants and the study’s first author (MF). The interviews lasted an average of 30 to 45 minutes.

#### The STS Model

In the study, the STS model was primarily used to develop the interview guide and analyze the data. It also provided a comprehensive understanding of the TSCP by addressing both its technical and organizational aspects. The STS framework integrates technical components, such as hardware and software, with social needs to ensure an effective human-computer interaction [28]. Within this framework, an STS aligns closely with an EHR, as its effectiveness depends not only on the technological infrastructure but also on how well it integrates with workflows, user needs, and the behaviors of individuals and organizations [28,29]. This perspective is supported by Coiera [30], who highlights that those systems that are reliant on complex human organizational structures, such as health care systems, are particularly well-suited to the STS approach. Moreover, Sittig and Singh [24] argue that models used for explaining technological systems are insufficient in capturing the full range of factors necessary for designing and implementing health care systems.

**Figure 2:**
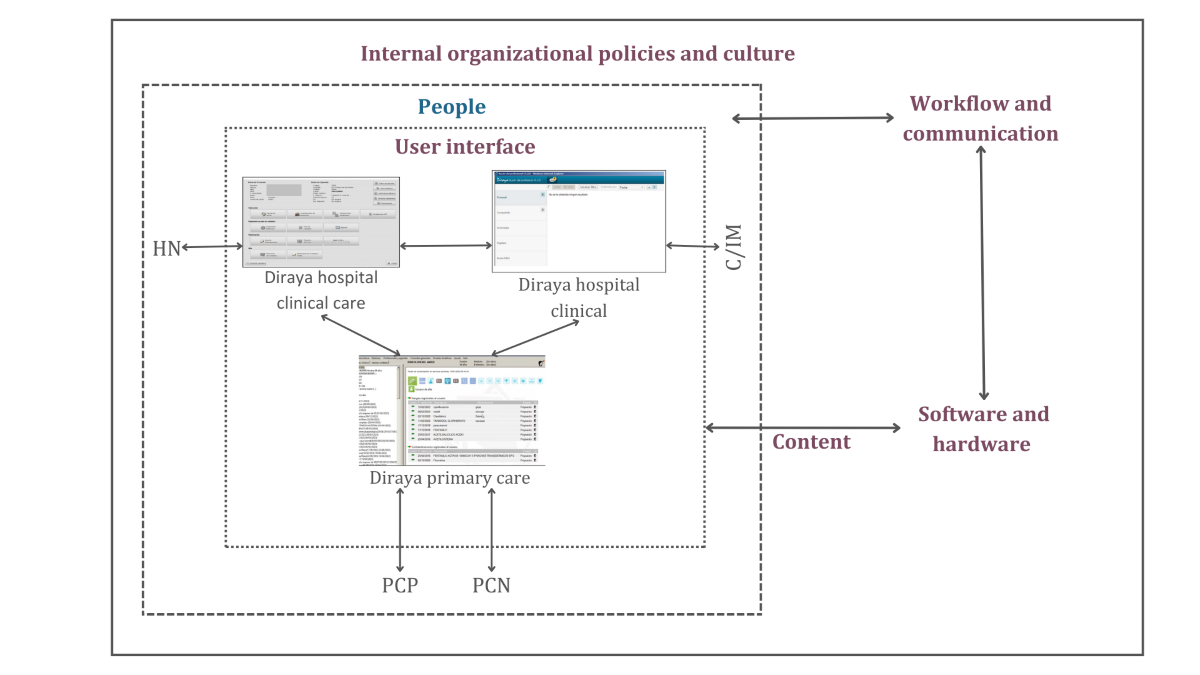
Interrelationships among the 6 dimensions of the sociotechnical model. C/IM: cardiologist or internal medicine; HN: hospital nurse; PCN: primary care nurse; PCP: primary care physician.

The STS model defines 8 dimensions [24], 5 of which were used in this study: clinical content, human-computer interface, hardware and software, workflow and communication, and internal organizational policies, procedures, and culture (Figure 2). The dimensions of external rules and regulations, as well as measurement and monitoring, were excluded, as they were less relevant to the 4 key actors in the study, given that clinicians are not directly familiar with these dimensions. The people dimension was also excluded because the direct interactions of HCPs with the system had already been analyzed before the interviews.

### Data Analysis

The data were analyzed using thematic analysis. After each interview, a preliminary analysis was conducted, followed by a comprehensive analysis at the end of the data collection. Initially, a deductive approach was used to identify the codes based on the 5 dimensions of the STS model. This was then followed by an inductive analysis to explore subthemes and themes in greater depth. As the interviews were conducted in Spanish, all quotes were translated into English. The data were organized in a Microsoft Excel spreadsheet, where the authors (MF and ND) familiarized themselves with the collected data, developed codes, and generated themes based on these codes [31,32]. On the basis of the data analysis, a list of improvements and recommendations was developed.

### Ethical Considerations

Although data collection was conducted across 6 health care areas within AHS, the Karolinska Institutet was the principal research institution. The study was carried out in accordance with the Swedish Ethical Review Act (SFS 2003:460) [33,34]. Since the research did not involve the handling of sensitive personal information as understood by the European General Data Protection Regulation, Regulation (EU) 2016/679 [35], ethics approval was not required. However, ethical requirements still apply. Candidates who expressed interest in participating received an informed consent form before the interview. In addition, participants recruited for the second phase signed an additional consent form. The informed consent form explained the study’s objectives, detailed how data would be handled to maintain anonymity, and clarified that the interview would be recorded and immediately transcribed. All participants were informed that their participation was voluntary and that they had the right to withdraw from the study at any time without any consequences. Additionally, no financial compensation was provided to any participants. Participants were given the opportunity to ask questions, and their understanding of the study was confirmed before proceeding. In addition, at the start of each Teams interview, participants provided oral consent before the recording began.

To ensure confidentiality and anonymity, only the study authors (MF and ND) had access to the data. A participant randomization document was created, listing names, demographic data, and participant codes. This document was stored in a password-protected Microsoft Excel database accessible only to the authors (MF and ND). The coded interview data were not linked to participants’ names. Antares Consulting, which is collaborating on the SCP implementation with Boehringer Ingelheim, shared nonsensitive and relevant data for the study, including participant contact information. In addition, during the interviews, 2 participants shared their screens to provide a clearer visualization of clinical history. Both participants used a fictitious profile, ensuring that the clinical history data did not belong to a real patient.

### Generative Artificial Intelligence Disclosure

In this study, generative artificial intelligence (AI) technologies, such as Grammarly (Grammarly Inc) and OpenAI’s ChatGPT (GPT-3.5; OpenAI), were used to help the authors (MF and ND) translate the quotes and documents from Spanish to English. These tools were not used to modify or influence qualitative data or the thematic analysis. The data collected were analyzed in their original form to ensure they accurately reflected participants’ responses. The tools were used in the preparation of the manuscript to enhance grammatical accuracy and clarity of the text. After using these tools, the authors (MF and ND) reviewed and edited all AI-generated content to ensure it aligned with the study’s objectives and maintained the integrity of the content. The authors (MF and ND) thoroughly reviewed and edited all aspects of grammatical accuracy and textual clarity to ensure alignment with the study’s objectives and preserve the integrity of the content, taking full responsibility for the publication’s content.

## Results

### Participants’ Demographic Data

Between January 15, 2024, and April 15, 2024, 30 HCPs were contacted, and 53% (n=16) agreed to participate in the study. While the goal was to recruit a balanced representation from each area, half (8/16, 50%) of the participants came from areas 3 and 4, resulting in lower representation from the remaining areas. Regarding professional roles, nurses made up 50% (8/16) of the participants, family doctors accounted for 25% (4/16), and nearly 20% (3/16) were cardiologists. As effective communication between different health care settings is essential for improving the transition of patients with HF to chronic care, an equal number of participants were selected from both primary care and hospital care. The detailed characteristics of the participants are presented in Table 2.

**Table 2:** Characteristics of study participants, including ID, age, clinical specialty, health care setting, and years of experience.

Participant ID	Age (y)	Clinical specialty	Health care setting	Experience (y)
PCP^a^ 1	30-40	Family and community medicine	Primary care	5-10
PCP 2	>60	Family and community medicine	Primary care	21-25
PCP 3	41-50	Family and community medicine	Primary care	21-25
PCP 4	51-60	Family and community medicine	Primary care	26-30
PCN^b^ 1	51-60	Nursing	Primary care	36-40
PCN 2^c^	41-50	Nursing	Primary care	26-30
PCN 3	30-40	Nursing	Primary care	11-15
PCN 4	30-40	Nursing	Primary care	11-15
HN^d^ 1	51-60	Nursing	Hospital care	36-40
HN 2^c^	30-40	Nursing	Hospital care	16-20
HN 3	30-40	Nursing	Hospital care	5-10
HN 4	41-50	Nursing	Hospital care	21-25
C/IM^e^ 1	51-60	Cardiologist	Hospital care	26-30
C/IM 2	51-60	Cardiologist	Hospital care	26-30
C/IM 3	51-60	Internal medicine	Hospital care	26-30
C/IM 4	30-40	Cardiologist	Hospital care	11-15

^a^PCP: primary care physician.

^b^PCN: primary care nurse.

^c^Participants corroborated the interviews after the analysis and proposed improvements.

^d^HN: hospital nurse.

^e^C/IM: cardiologist or internal medicine.

### Thematic Analysis

#### Overview

The results of the thematic analysis are presented in Table 3. The first column identifies the dimension of the STS, and the second and third columns list the subthemes and themes. In total, 65 codes, 23 subthemes, and 8 themes were identified. In addition, a matrix was developed to integrate the results with the actors.

**Table 3:** An overview of sociotechnical dimensions, subthemes, and themes.

Sociotechnical dimensions and themes			Subthemes
**Clinical content**			
	Optimizing medical data management for enhanced clinical workflow	Implementation of standardized clinical reports Reporting management challenges	
	Agreement on standardization and enhancement of the discharge report	Enhancement of the discharge nursing report Summarization of the medical discharge report	
	Enhancing clinical decision support through updated guidelines and automated tools	Nursing support to achieve better educational care Possibility of a medical decision support	
**Human-computer interaction**			
	Improving UX^a^ and usability for enhanced clinical efficiency and less time-consuming experience	Need for learnability Need to improve the visual design Noneffective iconography Nonefficiency interaction	
**Hardware and software computing infrastructure**			
	Enhancing technical infrastructure to improve HCP^b^ efficiency	Hardware infrastructure limitations Network connectivity Technical apparatus issues Software infrastructure challenges	
	Optimizing interoperability as a solution for better management of patients with heart failure	Unification of the EHR^c^ modules Streamlining processes with shared scales	
**Workflow and communication**			
	Encourage communication based on digital tools and personal connection	Inclusion of mailbox as a communication tool in workflow TSCP^d^ implementation workflow on EHR practices Fostering of effective internal communication Enhancement of external communication	
**Internal organizational policies, procedures, and culture**			
	Cultivating a culture in nursing practice and professional organizational integration	Willingness to improve the nursing care between primary care and hospital Training upon joining Cultivating culture	

^a^UX: user experience.

^b^HCP: health care professional.

^c^EHR: electronic health record.

^d^TSCP: transition standardized care pathway.

#### Clinical Content

##### Overview

**Figure 3:**
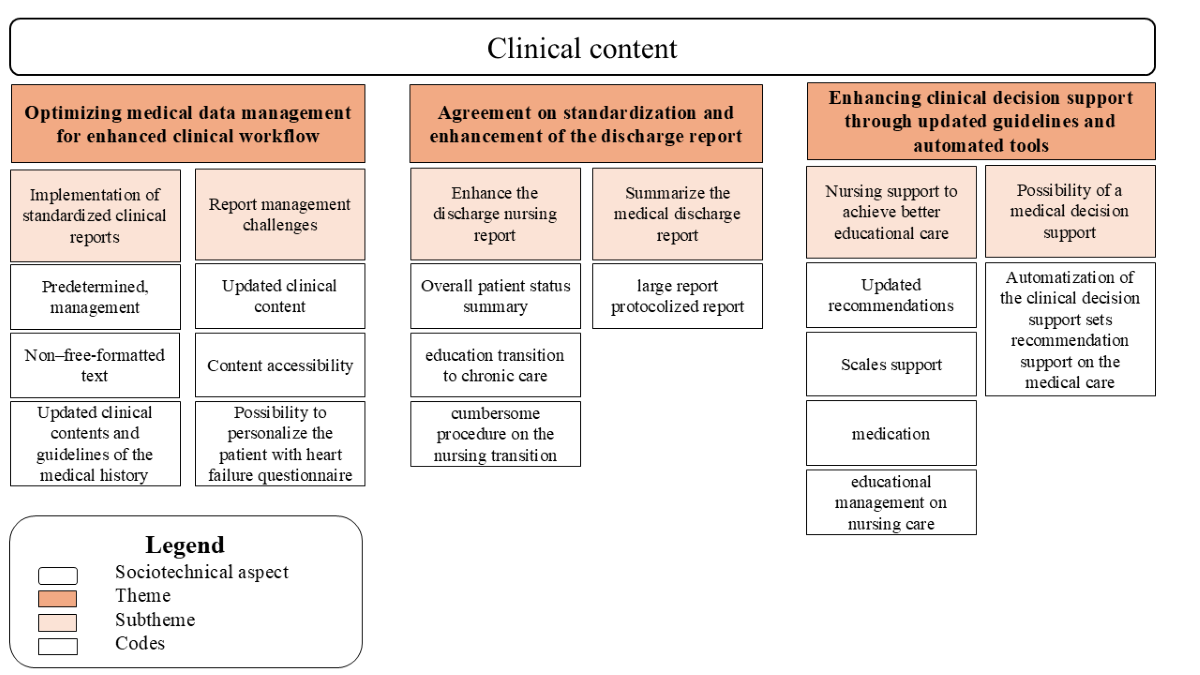
A hierarchical structure displaying the sociotechnical dimension, the codes, and the subtheme for themes 1, 2, and 3. CDS: clinical decision support; HF: heart failure.

Figure 3 illustrates the relationship between the sociotechnical dimension of clinical content, the codes, and subthemes related to the themes—optimizing medical management for enhanced clinical workflow, agreement on standardization and enhancement of the discharge report, and enhancing clinical decision support (CDS) through updated guidelines and automated tools.

##### Theme 1: Optimizing Medical Data Management for Enhanced Clinical Workflow

Primary care professionals, both in nursing and medical roles, are required to report each visit with a document that includes the patient’s medical history and relevant examination information. In AHS, after consultations with patients with HF, both PCPs and PCNs must complete the aforementioned report using 1 of the 2 available formats: either a regular clinical report or a report manager. Efficient management of the medical data collected during visits is crucial. Thus, this is facilitated by a protocol-based strategy designed to enhance clinical workflow. This theme explores the outcomes of both formats, addressing their clinical content as well as structural and technical usability aspects, especially in the context of HF practice.

One method of documenting involves using the clinical report, in which the medical history appears at the beginning of the report, followed by details of the physical examination. Nonetheless, variability exists in this format, as participants expressed the need for a standardized version. This approach would ensure consistency in both medical data and clinical content within the reports, particularly during the first visit after discharge. It would also minimize redundant data entry, saving time, and reducing the risks of confusion, misinformation, and errors. However, keeping the medical history up to date remains a challenge, as only physicians are authorized to make updates, and this task depends on their workload and willingness:

The medical history data is only updated when completed by the practitioner, leading to occasional information gaps...Sometimes, when a patient visits without a prior diagnosis of HF, I have to review the entire medical history to understand their condition.

PCN3

The alternative documentation format is through a report manager, which uses a questionnaire-style format detailing the actions required during a general HF visit. However, participant engagement with this tool is low, only 13% (1/8) of the participants had used it, while 63% (n=5) were unaware of its existence. Aside from increasing awareness of the report manager, the interviews identified several other needs and shortcomings. For example, participants recommended that both data entries and questions remain up to date and relevant, while also being user-friendly and requiring minimal effort to complete:

The content sometimes contains inaccuracies or is not aligned with the requirements of the visit...I need the content to be current...some scales that are included are not typically performed in primary care and should only be conducted in the hospital.

PCP1

Thus, it was also suggested that each questionnaire’s content be specifically tailored to the professional’s roles and the type of care they provide. Finally, all clinical content is currently saved as plain text, which limits navigation. Therefore, professionals expressed a preference for a more structured format:

When the Report Manager is filled out, the information is directly transferred to the clinical record, where it appears overly condensed and difficult to read. I would prefer the information to be displayed in bullet points, following the same sections as the Report Manager.

PCP1

##### Theme 2: Agreement on Standardization and Enhancement of the Discharge Report

To identify the needs and areas for improvement in the nursing discharge report (NDR), both HN and PCN were asked specific questions. As HCPs from both settings, their collaboration helped pinpoint which parameters could be optimized.

Focusing first on PCN, they revealed that they never review the NDR because the information appears “copied and pasted” and lacks patient-specific details. Therefore, PCNs used to read the medical discharge report (MDR) instead:

Well, I’ll tell you that when they discharge a patient, the nurse fills out a form that nobody really reads. Are these responses to a questionnaire about what has happened to the patient? How does he sleep? Does he have any risks? How is his ulcer treated? How is he catheterized? .... I have too many questions that are not provided and others that are unnecessary. If the patient is not catheterized, I don’t need to know that he doesn’t have a catheter; it’s only relevant if he does...

PCN 3

Following up with HNs, professionals confirmed that the information received from PCNs is often incomplete. HNs often do not have time to complete the NDR, and if they do, it tends to be very broad and not personalized. They would like to have parameters, such as social risk, patient polypharmacy, fall risk, Barthel scale, edema, weight at discharge, oxygen therapy, dyspnea, and feeding, among other options. This would provide PCNs with a summarized and relevant report of the status of patient with HF. Moreover, it would enhance communication between both health settings:

Patient education and management of medication and alarm signs are essential information that must be worked on in both settings. But knowing how these are being addressed and what the patient’s status is from PC and in HC is essential so [that] it can be agreed upon and worked on together.

PCN 3

In contrast, PCPs commented that the MDR is overly extensive and tedious, requiring a significant amount of time to thoroughly review the details. They suggested that a more concise report, with specific parameters, would be beneficial. This should include key information, such as the confirmation of the initial diagnosis, etiology of the condition, left ventricular ejection fraction data, potassium levels, creatinine levels, and the treatment plan of the patient, including therapies and doses.

##### Theme 3: Enhancing CDS Through Updated Guidelines and Automated Tools

Although CDS features exist in the current Diraya system, they are limited to nursing professionals. In the Diraya primary care recommendations based on the responses are provided upon completing a questionnaire. Nonetheless, PCNs have expressed concerns regarding the clinical content and functionality of the CDS:

It will be nice to have recommendations together with the scale result, the associated risks, and how to manage the patient. I usually remember from experience, but it’s harder to recall after some days off.

PCN3

In addition, managing medication for patients with HF is crucial for ensuring adherence to treatment and reducing readmissions. PCNs would find it beneficial if medication information included its purpose and use instructions:

Sometimes, the treatment involving medication reconciliation causes certain confusions: medications are added without educating the patient. Occasionally, medication is duplicated, and the primary care physician suggests resolving it with the hospital’s internal medicine department.

PCN1

A key component of the TSCP is patient education after hospital discharge through telephone or on-site visits. In addition to providing medication information, it also involves informing patients about their illness, warning signs, and associated risks according to HN and PCNs having a checklist including this information would be helpful:

Having a checklist with all the necessary information would ensure nothing is overlooked.

HN2

Regarding the report manager, the CDS provides prompts based on questionnaire results. However, these prompts are in free text and do not link directly to actions in the EHR. Automating the CDS to enhance patient management and streamline administrative tasks would be beneficial. In contrast, PCPs do not receive any system-generated recommendations during their visits. Addressing this gap is essential to improving their experience, so feedback and needs were collected:

It would be incredible if the system could guide me based on protocols and guidelines for HF patients, such as in scales and patient examinations. This would enable me to make more informed decisions.

PCP 2

Finally, it is also important to mention the integration of CDS during the TSCP while the patient is hospitalized. An HN suggested that including recommendations for assessing scales used at the time of discharge would be helpful. Similarly, C/IM specialists mentioned that CDS could provide valuable guidelines and recommendations for patient evaluations during follow-up visits, potentially improving overall care practices.

#### Human-Computer Interaction

##### Overview

**Figure 4:**
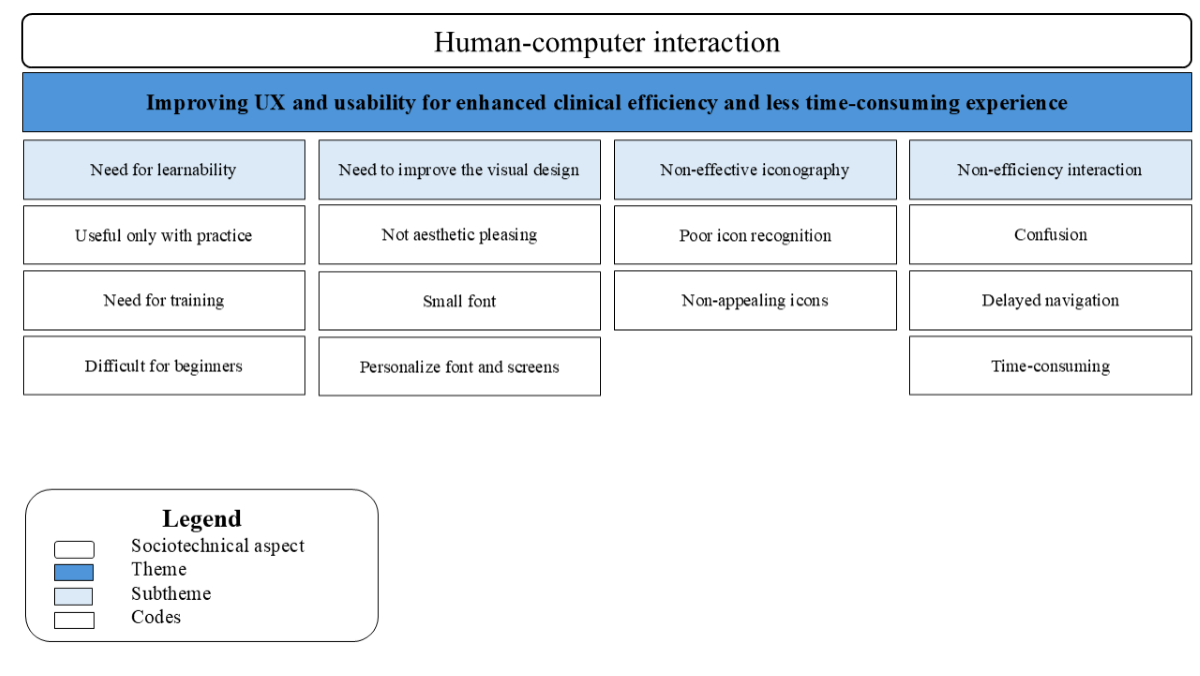
A hierarchical structure displaying the sociotechnical dimension, the codes, and the subtheme for theme 4. UX: user experience.

The interaction among the 4 groups of interface users varied depending on the health care setting and the professional role. PCPs used one module of the interface, while hospital users used 2 different modules, and primary care HCPs only had access to one module. Although these 3 modules differed, participants shared similar needs and insights. Figure 4 illustrates the relationship between the sociotechnical dimension of human-computer interaction, the codes, and subthemes related to the theme—improving user experience (UX) and usability for enhanced clinical efficiency and time-consuming experience.

##### Theme 4: Improving UX and Usability for Enhanced Clinical Efficiency and Time-Consuming Experiences

All HCPs agreed that they gradually became proficient in using the EHR for HF care through practice, indicating that the interface is not intuitive. Some found it challenging at first, though 1 PCN mentioned that it became manageable after gaining experience:

For me, once learned, its [the EHR] operation was straightforward.

PCN4

Professionals also emphasized the need for adjustable font sizes to meet various needs. They suggested that visual enhancements could streamline information retrieval, ultimately improving attention and efficiency.

Moreover, HN explained completing the NDR involves navigating numerous pages, making system interaction cumbersome and the design unappealing. Notably, C/IM specialists reported greater satisfaction with the interface compared to PCPs.

All PCPs commented that the interface icons, particularly those used for medical history, were unclear:

For patients with COPD, a lung icon appears; for cervical cancer, there is a figure of a woman; and for HF, a heart icon is used. However, it is not immediately clear whether the icon represents HF. I would use a clearer icon, or better yet, the diagnosis in text form.

PCP2

Some (5/8, 63%) participants noted that the icons used throughout the questionnaire were difficult to understand:

You need to recognize that one icon represents nursing and another medicine; others stand for medication, vaccine, diagnosis, video call, and more. But with no text in line with the icons, their meanings are unclear.

PCN 3

Regarding access to EHRs across different health care settings, hospital participants reported rarely having time to consult the primary care EHR. Conversely, primary care participants often find time, express interest in learning more about hospital data, or value the patient, which motivates them to review the hospital EHR. However, they emphasized the difficulty of doing this regularly or systematically due to time constraints:

There is no time and accessing the hospital EHR is time-consuming; it can take up to five minutes to find specific information, which wastes quality time with the patient.

PCN2

Therefore, many HCPs choose not to access the data regularly, disrupting communication between professionals.

#### Hardware and Software Infrastructure

##### Overview

**Figure 5:**
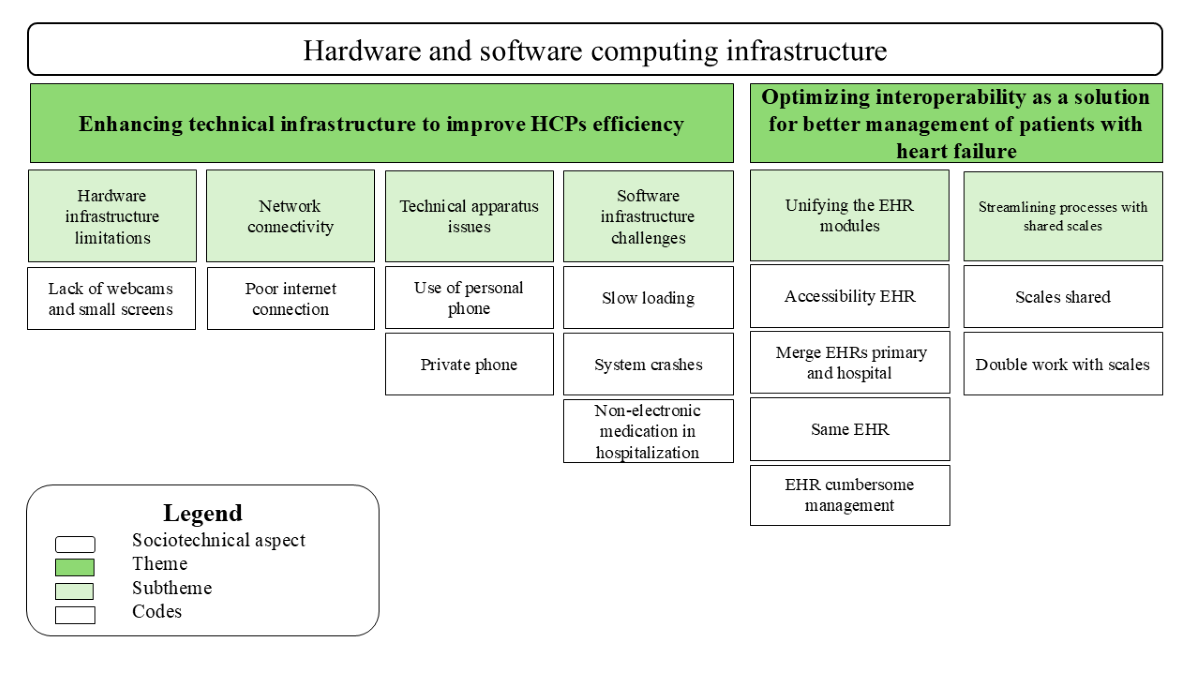
A hierarchical structure displaying the sociotechnical dimension, the codes, and the subtheme for themes 5 and 6. EHR: electronic health record; HCP: health care professional; HF: heart failure.

In this dimension, the findings indicated that the technology used in primary care centers and hospitals was of low quality, resulting in significant delays and extended waiting times. Moreover, interoperability played a vital role in connecting both settings, which was crucial for the effective management of patient care during the discharge transition. Figure 5 illustrates the relationship between the sociotechnical dimension of hardware and software computing infrastructure, the codes, and subthemes related to the themes—improving technical aspects reduces unnecessary task time, thereby enhancing the quality of work; and optimizing interoperability as a solution for better management of patients with HF.

##### Theme 5: Improving Technical Aspects Reduces Unnecessary Task Time, Thereby Enhancing the Quality of Work

Most of the participants highlighted the inefficiency of current technology, including hardware, software, and internet connectivity—used in their health care settings. They reported frequent internet outages, especially during rainfall, along with system crashes and slow loading times when using the Diraya system. Participants working in hospitals also faced malfunctioning computers, which caused significant delays in accessing necessary documentation tools:

The internet goes down at least three times a week. [C/IM 3])

Another issue was the use of corporate phones with long and unrecognizable numbers, which affected the availability of PCNs for patient follow-ups within 48 hours to 72 hours after discharge as patients often ignored these numbers:

Patients frequently disregard calls from unknown, lengthy numbers, leading to missed follow-up opportunities.... Providing patients with recognizable extension numbers would greatly enhance communication. [PCN 2])

In addition, some HCPs do not have access to corporate phones, forcing them to use their personal devices for work-related communication. This raises several concerns, such as the traceability of patient interactions and data privacy:

I have a friend who works in the cardiology department of another hospital. When I know a patient is from her area, I use WhatsApp to inform her...which means I take work home and there’s no traceability for these patient interactions.

HN 4

This issue extends even to professionals within the same health care center. Some PCNs mentioned that informal communication methods, such as WhatsApp messages or post-it notes, are used to arrange extra notes for patients with HF after discharge. These practices highlight the need for a more structured and reliable communication system to ensure patient care effectively.

##### Theme 6: Optimizing Interoperability as a Solution for Better Management of Patients With HF

To provide appropriate care for patients with HF during hospital discharge, it is essential that medical reports are saved and accessible across different health care settings. The single health record allows HCPs to view comprehensive patient reports across facilities. However, many professionals expressed dissatisfaction with its interoperability.

PCPs often struggle to access important clinical discharge reports or test results because they are either not uploaded or incomplete:

From the Single Health Record, I often cannot access the discharge report from medicine or nursing...it is either not uploaded, or there are tests that are not included in the report, so I have to review the hospital EHR, which takes a lot of time.

PCP 2

This might be due to a lack of awareness among HCPs regarding the correct process for adding information:

For the nursing report to be uploaded correctly, you need to click on an icon, and many times professionals are not aware of this.

HN2

Some PCPs mentioned that they access the hospital’s single health record only when necessary due to the cumbersome and time-consuming process. Ideally, this should be regular practice to ensure continuity of care. Within hospital settings, C/IM professionals also echoed this concern, emphasizing the importance of seamless integration between primary care and hospital systems to facilitate smoother patient care transitions:

At the time of hospital discharge for heart failure patients, having all the information from primary care is crucial during the initial visit.

C/IM 4

#### Workflow and Communication

##### Overview

**Figure 6:**
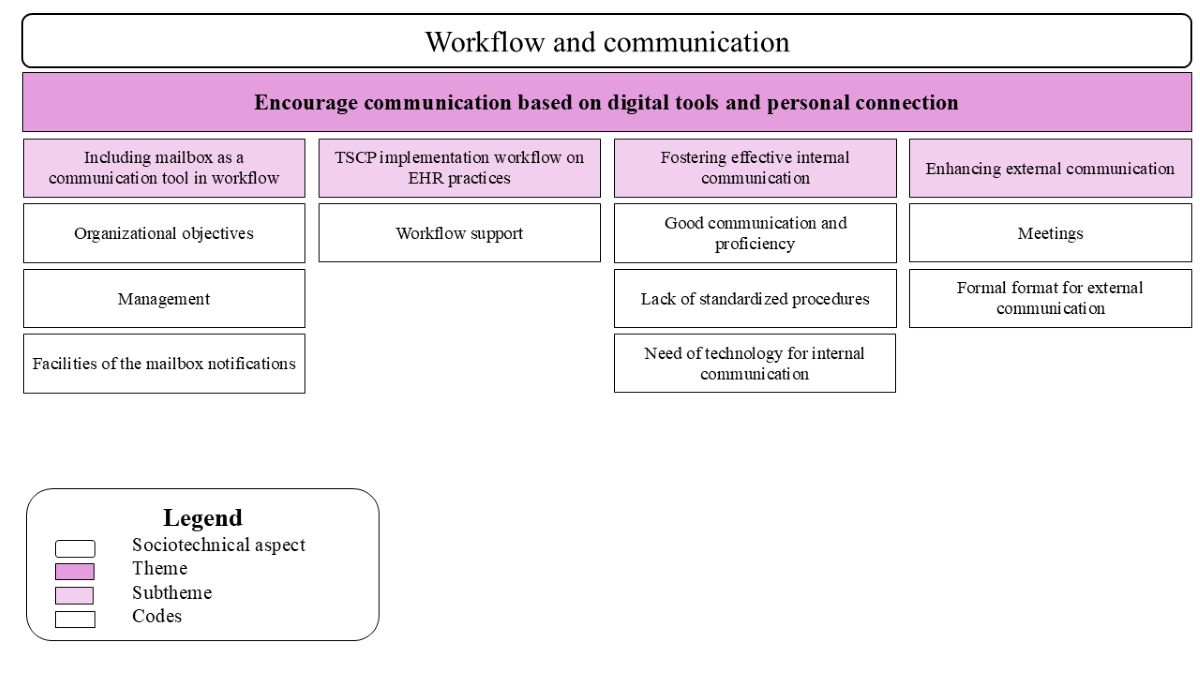
A hierarchical structure displaying the sociotechnical dimension, the codes, and the subtheme for theme 7. EHR: electronic health record; TSCP: transition standardized care pathway.

As previously stated, during the interviews, the importance of communication between different settings and professionals was clear, emphasizing its crucial role in ensuring a smooth transition from acute to chronic care. Data analysis showed that communication through EHR can take various forms—internal, external, and both direct and indirect. Internal communication occurs among HCPs within the same health care setting and can be either direct (eg, face-to-face conversations and meetings) or indirect (eg, through the EHR and messages). In contrast, external communication occurs between HCPs from different health care settings and can also be either direct (eg, seminars) or indirect (eg, via the Diraya mailbox or interconsultations). Consequently, participants identified several needs and suggested improvements for these different forms of communication. Figure 6 illustrates the relationship between the sociotechnical dimension of workflow and communication, the codes, and subthemes for the theme—encourage communication based on digital tools.

##### Theme 7: Encourage Communication Based on Digital Tools

Regarding internal communication, participants mentioned that it is usually informal yet effective and efficient in most cases. Some centers explained that to communicate with a colleague, they often leave a note, send a WhatsApp message, or speak directly to them. Nonetheless, other participants explained that storing sensitive information in a written note or spoken conversation is not the safest approach for medical care. Moreover, using personal phones to store confidential information is a concern:

I usually knock on the door or I leave the information on a post-it.... It would be useful to have an integrated communication and chat system, as currently, we communicate informally.... It would be beneficial if the information were automatically transmitted through the system.

PCN4

In contrast, some participants emphasized that certain primary care centers are quite familiar with their current process and feel no technological innovation is necessary. They rely on notes in clinical history for another professional to view, and this approach usually works well:

In my opinion, within our field, I would not recommend any technological aid because it could lead to a loss of personalization. As I have the nurse next door, I would not like to have a chat.

PCP4

Some PCPs mentioned that they share schedules with PCNs to discuss patients, but these schedules are rarely used.

Within the hospital system, 50% (2/4) HNs indicated that communication with the medical department during hospital discharge needs improvement. A major issue is that, often, when a patient is discharged, the nurse is not informed by the physician, which sometimes causes delays in completing the discharge report for patients with HF:

Sometimes, when the patient is discharged, I am not informed. Although communication with colleagues is good, sometimes the workload causes a noneffective communication.

HN2

Regarding communications between HCPs from different medical settings, known as external communications, participants noted that direct communication between physicians from both settings is more established and standardized than communication between nurses across these contexts. However, one participant pointed out:

In my center, we maintain a very fluid relationship because I know the head of the cardiology department at the hospital; however, it is informal communication. In other centers or specialties, this type of one-way communication does not exist.

PCP 3

Nevertheless, the same participant added:

The idea of conducting clinical sessions is excellent and useful, but it requires time and resources that are unavailable.

PCP 3

In addition, from the primary care perspective, there is a recognized need to establish a bidirectional communication channel with cardiologists, as information is sometimes lost. From the hospital’s medical site, C/IM departments commented that communication is sufficient, with improvements mainly needed in document interoperability. They also pointed out that in some centers, teleconsultations between PCPs and C/IM staff are very successful and efficient.

In contrast, nursing professionals all agreed on the need to enhance direct communication between nursing professionals from both settings. They emphasized the importance of workshops or sessions to standardize patient care and better understand nursing practices across settings:

I would like to hold sessions with PCNs to learn about what they do and how I can better emphasize patient care. Sometimes I send them information, but I think they do not consider it important. I would like to meet the PCNs to become more efficient in managing the patient during the transition. For example, educating the patient about medication for HF is very important, and I would like to know how they do it so I can determine what information I need to provide at the time of discharge.

HN3

One communication method in place is the mailbox module, which sends notifications to primary care professionals when a patient’s status changes, such as when a patient is discharged from the hospital. For patients with HF following the TSCP, when the patient is discharged, both the PCP and PCN receive a notification that includes the patient’s name, medical history, and the reason for the previous hospitalization. If no follow-up appointment has been scheduled, the nurse must call the patient within 48 hours to 72 hours and visit them at the primary care center. Even though the protocol was clear, several shortcomings and needs in this communication method were identified during the interviews.

Several PCPs expressed that upon receiving this alert, they need access to more comprehensive patient information:

When I receive an alert and the reason for admission is not heart failure but another condition that refers to heart failure, ... I do not call the patient or review the medical history. As a result, this patient is overlooked.... If I had more time and better organizational support, I could handle it more effectively.

PCN 4

In contrast, the hospital lacks a Diraya mailbox module, thus discontinuing the communication from primary care. Among professionals, interconsultations through Diraya between physicians at primary care and hospitals are commonly used, which are generally effective according to the participants. However, for nursing staff, such interconsultations are not available. Consequently, nurses have expressed the desire to implement such interconsultations between both settings, when necessary, to enhance the management of patients with HF at discharge.

In primary care, HCPs are accustomed to managing visits for patients with HF. However, when using Diraya, the purpose of each visit often remains unclear. To improve care during the transition to discharge, professionals need to have a clear guide that outlines the specific tasks for each visit. Implementing an HF guide or protocol within the system would streamline these visits, enabling HCPs to effectively adhere to the SCP:

I would appreciate having instructions to understand the patient’s status and which post-discharge visit they’re attending.... All this information is protocolized, but we don’t see it; we have to know it by heart.

PCN2

#### Internal Organizational Policies, Procedures, and Culture

##### Overview

**Figure 7:**
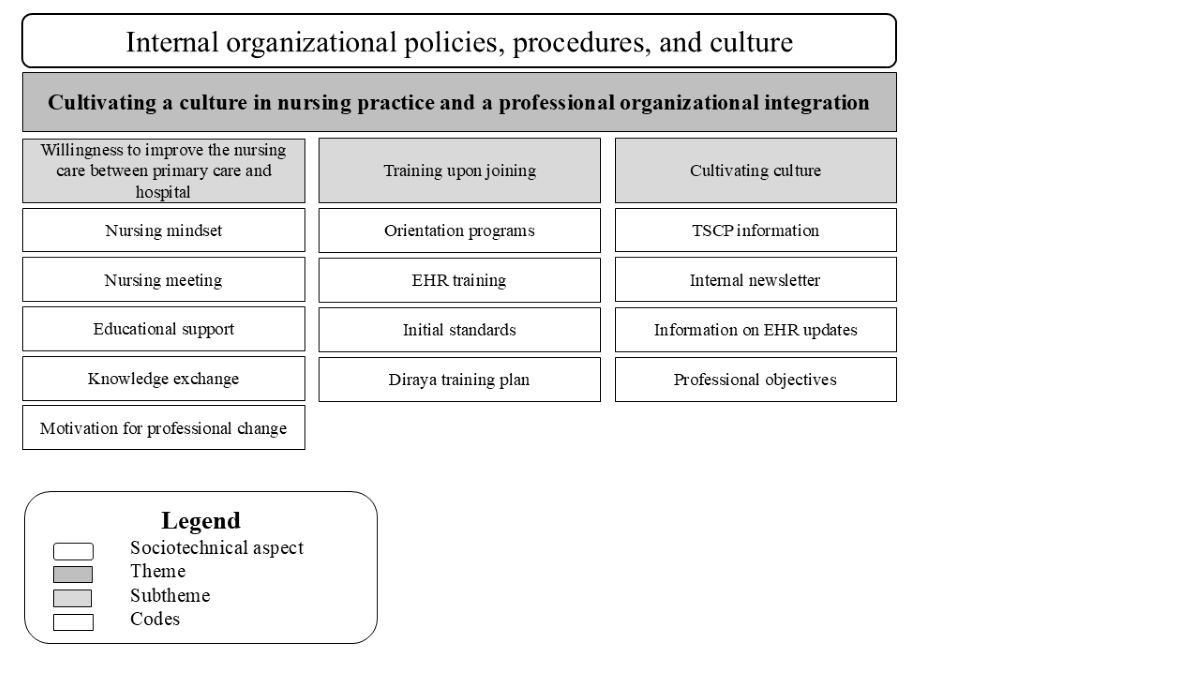
A hierarchical structure displaying the sociotechnical dimension, the codes, and the subtheme for theme 8. EHR: electronic health record; TSCP: transition standardized care pathway.

Figure 7 illustrates the relationship among the sociotechnical dimensions of internal organizational policies, procedures, and culture; the codes; and the subthemes for the theme—cultivating a culture in nursing practice and professional organizational integration.

##### Theme 8: Cultivating a Culture in Nursing Practice and a Professional Organizational Integration

Nurses were interested in taking on a more empowered role and increasing their involvement in decision-making during the patient transition to a higher level of care:

I would like the cardiology specialist nurses to provide us with training, so we can gain more knowledge.

PCN2

Indirectly, all of them suggested the importance of collaboration to facilitate cultural change between hospitals and primary care settings:

I am very eager to undertake projects to improve patient education for those with HF.... I look forward to meeting the primary care nurses who treat these patients to understand what they do.

HN4

Participants reported no organizational dissatisfaction regarding the implementation of the TSCP. However, they identified a potential incentive for HCPs: incorporating TSCP-related tasks into primary care employment objectives. This approach would encourage staff participation and enable verification of each HCP’s work.

Some participants expressed the need for structured training on Diraya when starting a new job, recognizing it as crucial for onboarding. However, they noted that HCPs often join on short notice, leaving no time for formal training. Others believed formal training was unnecessary, stating:

It’s not necessary; you learn on the job. If it’s an optional course, no one will take it. What is needed is support during your initial days.

C/IM2

In addition, some professionals mentioned that at AHS, Diraya training sessions are available and can be completed in their free time:

At AHS, there are straightforward and accessible Diraya courses, but we have to complete them at home. Ideally, these should be part of work hours or count towards points in the employment pool.

HN2

Finally, 3 participants noted that they receive little information about organizational updates, which they consider crucial.

They expressed a desire for managers to keep them better informed about improvements.

#### Suggestions for Improvements Based on the Results

On the basis of the needs identified in the interviews, several improvements and recommendations have been proposed. These were validated by additional interviews with 2 participants, HN2 and PCP2. A summary of the proposed improvements and suggested actions, linking them to the corresponding key findings in each theme and the HCPs involved in the TSCP, can be found in Multimedia Appendix 2. In addition, each improvement was assigned to a priority scale, which was corroborated by HN2 and PCP2. The priority levels were (1) not important—enhances HCP performance but is not critical for managing patients with HF within the TSCP; (2) important—optimizes HCP workflow and is important for managing patients with HF within the TSCP, though not crucial for its implementation; and (3) urgent—crucial for optimizing HCP workflow and urgently needed for managing patients with HF within the TSCP, as its absence prevents the correct implementation of the TSCP.

#### Proposed Implementation Plan

**Figure 8:**
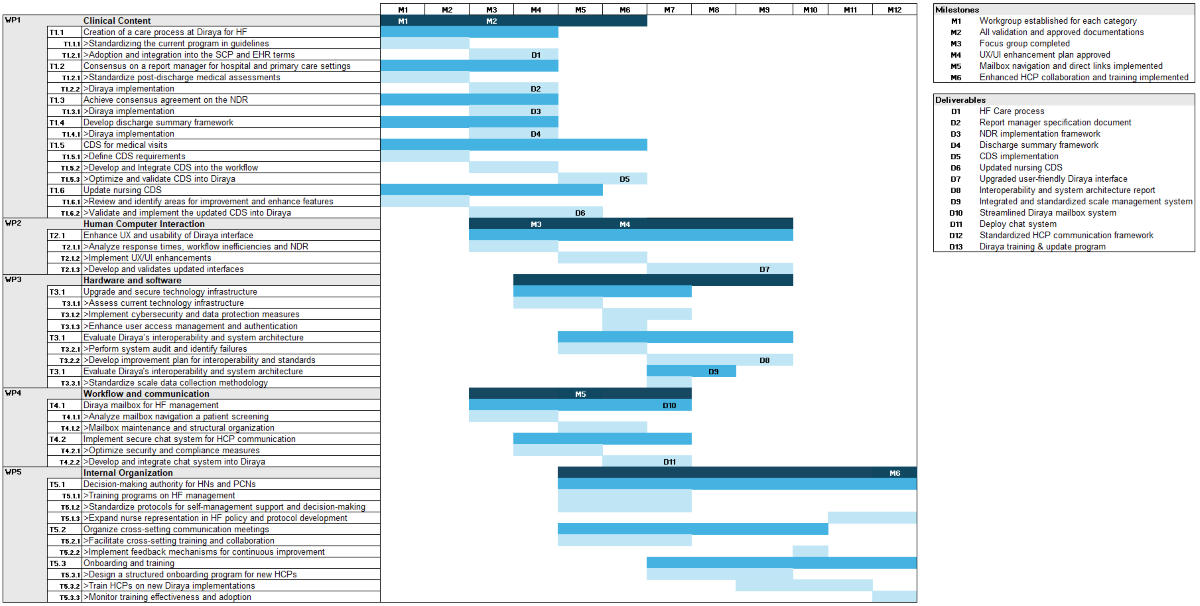
Suggestion for a detailed implementation plan. CDS: clinical decision support; EHR: electronic health record; HCP: health care professionals; HF: heart failure; HN: hospital nurse; NDR: nursing discharge report; PCN: primary care nurse; SCP: standardized care pathway; UX/UI: user experience/user interface.

Engaging diverse stakeholders, including IT teams, policy makers, and patients, is crucial for the successful optimization and implementation of EHRs and it is also an aspect of the implementation process of the MAIC toolkit. Strategies such as structured communication channels, collaborative platforms, advisory committees, training sessions, and feedback systems can facilitate this engagement. Establishing consistent communication with different stakeholders can identify and address potential barriers during the implementation. In addition, forming committees with representatives from each stakeholder group ensures diverse perspectives are considered. To optimize and implement improvements, incorporating a Gantt chart into the implementation process is recommended. In Figure 8, each work package represents a dimension of the STS, with tasks correlated to the improvements in Multimedia Appendix 2. A 1-year implementation plan is suggested, based on the progression of tasks and subtasks, and should be tailored to the available resources of the health care system. The proposal includes conducting training sessions and regular meetings to align expectations and identify needs. Specific working groups will be established to enhance communication and task execution. In addition, focus groups with end users are proposed to gather feedback and adjust the implementation according to their needs.

## Discussion

### Principal Results and Comparison With Previous Work

This study identified the needs of HCPs when implementing the transition standardized care model, MAIC [16], for HF practice within the Diraya system. In addition, it provided recommendations for optimizing the EHR within this context. Notably, the HCPs involved in this study include PCPs, PCNs, HN, and C/IM specialists.

The results showed that the Diraya system does not adequately support the implementation of the TSCP. HCPs are required to complete many unnecessary tasks, ultimately increasing their workload. In addition, these tasks are often ethically inappropriate and add to the administrative burden. The findings also suggested that to implement the TSCP more efficiently, changes and improvements are needed in clinical information within Diraya, the computer system, and the AHS organization.

The first theme, *optimizing medical data management to enhance clinical workflow*, primarily addressed in primary care settings during the initial visits after discharge, suggested the creation of a specific care process for HF in Diraya, as well as achieving a committee consensus on a report manager. The findings highlighted that, as part of the EHR optimization, it is crucial to ensure smooth clinical workflow and personalized data exploration [36]. These 2 improvements, considered high priority, aligned with studies emphasizing the optimization of data management as a key initial requirement [37]. In addition, other studies showed that customizing report managers and care processes for each EHR helps with accurate patient evaluation and effective data analysis. This underscores the need for HCPs to collect and analyze health indicators to better understand the disease state [38,39]. Moreover, customizing the EHR through consensus among various HCPs is consistent with current literature, which underscores the importance of incorporating quality standards in the EHR development through HCP collaboration [21].

As discussed, in the second theme, *agreement on standardization and enhancement of the discharge report* is recommended to create a consensus document for both the NDR and a discharge summary for the MDR. This would improve communication between health care settings and ensure compliance with the proposed TSCP. The findings highlighted 2 key aspects. First, they identify technological barriers to implementing an automation tool in the EHR system—an essential feature for managing HCPs’ workload but also a challenge for the software [40]. Therefore, it is crucial to ensure that the tool is intuitive and user-friendly for HCPs before implementation. Second, the results aligned with studies emphasizing the importance of a nursing care plan during the discharge process to enhance patient care [39].

Regarding the third theme, *enhancing clinical decision support through updated guidelines and automated tools*, it was proposed to implement CDS tools for both medical visits and to update the nursing CDS, including the NDR and the PCN report manager. This would ensure proper compliance with the TSCP during patient visits and provide essential support to HCPs across different health care settings. This recommendation aligns with studies suggesting that effectively integrating CDS tools reduces the cognitive burden on HCPs, enhances clinical decision-making, and leads to better patient outcomes [41]. In addition, research on HF has shown that comprehensive CDS tools significantly reduce hospital readmissions and improve adherence to treatment protocols, such as TSCP [42,43]. Therefore, updating the nursing CDS and implementing CDS tools in medical visits is crucial.

Regarding the fourth theme, *improving UX and usability for enhanced clinical efficiency*, the study highlights how the interface design subtly influences daily procedures and, over time, impacts the workflow of HCPs managing HF practices. To address interface limitations, 2 key recommendations are proposed. The first and most urgent step is to improve the iconography of specific HF-related symbols and the text size. Once this is achieved, the focus should shift to broader improvements across the entire interface to enhance overall usability. These changes are essential, as studies show that complex or poorly designed EHR systems can create stress and pressure on HCPs, negatively affecting their daily work. Enhancing these aspects would directly support HCPs in efficiently performing TSCP-related tasks [44].

The findings from theme 5, *enhancing technical infrastructure to improve HCPs* efficiency, highlighted several critical needs and shortcomings that must be addressed to efficiently implement the TSCP. These include improvements in both hardware and software, network connectivity, and technical equipment. In addition, evaluating the cybersecurity infrastructure is essential, as the system stores sensitive data, and HCPs often resort to using private resources due to issues, such as low connectivity. These findings align with previous research indicating that inadequate technical infrastructure is a major barrier to implementing an SCP in specific health care settings [45].

Regarding theme 6, *optimizing interoperability as a solution for better management of patients with HF*, the need for improved interoperability emerged consistently throughout all interviews. To address this, it is recommended to evaluate the system’s current standards and protocols and integrate common assessment scales across health care settings. This would enable better patient monitoring and communication between hospitals and primary care. These findings align with studies emphasizing the importance of sharing documents across different health care settings to improve communication [39].

The interoperability challenges, usability concerns, and workflow inefficiencies in the current EHR system in general are global challenges. The World Health Organization (WHO) emphasizes the need for standardized data exchange and interoperability to ensure seamless health information flow across different systems and regions [46]. They also highlight the importance of user-friendly digital health solutions and the necessity of optimizing digital health tools to support efficient workflow. Several best practices have been established to address these issues. WHO emphasized that digital interventions for health systems should leverage standardized health data exchange formats [47]. For achieving interoperability among systems, standards, such as HL7 Fast Healthcare Interoperability Resources, and terminologies like International Classification of Disease, Systematized Nomenclature of Medicine Clinical Terms, and Logical Observation Identifiers, Names, and Codes are crucial for the seamless integration of EHR systems [48-50]. The WHO Digital Health Guidelines emphasize human-centered design principles to improve EHR usability, ensuring intuitive interfaces and role-specific workflows [47].

Regarding theme 7, *encouraging communication based on digital tools and personal activities*, effective communication is the cornerstone of this study. In an SCP dedicated to discharge and the transition to primary care, clear and efficient communication is essential [51]. Many of the other identified challenges within Diraya are also linked to communication barriers. Research confirms that poor communication is a common issue, and overcoming it requires stronger relationships between HCPs to facilitate referral processes [52]. In addition, studies have shown that electronic communication between primary care and other health care settings is both feasible and beneficial, as it enhances accuracy in information sharing [53]. However, it is crucial to address the privacy concerns surrounding the use of informal communication tools, such as WhatsApp and handwritten notes, particularly regarding data security and compliance with local privacy regulations. Informal communication tools may not provide the necessary safeguards to protect patient information, potentially leading to violation of confidentiality and noncompliance with regulations like the General Data Protection Regulation and the Health Insurance Portability and Accountability Act [54,55]. Ensuring that all communication methods adhere to stringent data security standards is vital to maintaining patient trust and safeguarding sensitive health information. On the basis of the results of this study, the authors (MF and ND) suggested implementing a chat system for internal and external communication among HCPs, ensuring timely mailbox updates, and organizing meetings and training sessions to improve external communication between both settings.

Finally, theme 9, *cultivating a culture in nursing practice and a professional organizational integration*, emphasized the need to strengthen HF care by empowering nurses in their daily work. Suggested strategies include providing seminars, educational programs, and decision-making assessments within the TSCP tasks. These improvements directly relate to the importance of designing an EHR system that serves both nurses and physicians equally. Current literature supports the idea that nurses must have a significant role in shaping digital tools like EHRs to ensure their smooth integration into patient care workflows. Several studies have demonstrated the positive impact of nurse-led interventions in transitional HF care. Nurse-coordinated transitioning of care significantly reduces readmission rates for patients with HF. Nurse-led transitional care interventions decrease health care use, including all-cause and HF-specific readmissions. These interventions also reduce mortality and improve psychosocial outcomes, such as health-related quality of life and self-care behaviors. In addition, nurse-led transitional care programs improve readmission rates, self-efficacy, functional status, and quality of life among patients with heart disease [56-59].

Nurses bring valuable clinical insights, which are critical for developing technologies that support decision-making and improve care delivery. However, challenges, such as excessive documentation, inefficient workflows, and poor system interoperability, can contribute to nurse burnout and affect care quality. To address these issues, the National Academy of Medicine recommended improvements to EHRs and related technologies to better support nurses and ultimately enhance patient care outcomes [60].

The findings of this study could be applicable to other regions in Spain for managing HF or other chronic conditions requiring coordination between primary care and hospital services. This is particularly relevant as the transition from hospital discharge to primary care in Spain faces significant challenges due to poor interoperability among EHR systems. This fragmentation hinders effective communication and continuity of care across different levels of the health care system. A study [61] highlights that one of the main issues in the SNHS is the lack of interoperability in the EHR systems, which negatively impacts communication between primary and specialized care. For example, in the principality of Asturias, a digital tool has been introduced to assess adherence to HF clinical practice guidelines within internal medicine departments. Hospitals also have a clinical protocol for managing and following up on HF. In addition, the comprehensive continuous care program for patients with HF has been implemented in 5 out of 62 nationwide units. The systems in use include Selene, Millennium, Catalonia primary clinical station, and others, causing a nonunified EHR system [62].

Our findings align with existing research emphasizing the critical role of sociotechnical factors in the successful implementation of EHR systems. By examining both social and technical factors, potential barriers and adoption, the risk of nonadoption or abandonment, and the impact of stakeholders, including patients and HCPs, can be identified [63]. Adhistya et al [64] highlight the critical role of a sociotechnical framework in the implementation of electronic medical records to enhance patient safety. Their research identifies those factors such as user behavior, system design, and the availability of supporting infrastructure influence patient safety outcomes [64].

### Limitations of the Study

While the qualitative approach using semistructured interviews and thematic analysis is appropriate, the sample size of 16 participants and the focus on specific areas within Andalusia may limit the generalizability of our findings.

To improve the generalizability of the results, future research should include a more extensive and diverse participant base. This could involve expanding the sample size and including HCPs from various regions and settings, such as rural and urban areas, and different hospital levels. In addition, incorporating perspectives from other stakeholders, such as IT professionals, patients with HF, administrative staff, and pharmacists, would provide a more comprehensive understanding of the challenges and opportunities for EHR optimization. In addition, a dedicated study group on case manager nurses is needed to explore their specific roles within the TSCP in greater detail. Exploring the impact of different socioeconomic and cultural aspects on EHR adoption and use would further enhance the applicability of the findings. Another limitation of this study was time constraints, which allowed for corroboration and validation of findings with only 2 participants, which was insufficient given the initial sample size. Future research addressing this limitation is necessary.

### Conclusions

Effective communication is essential for managing patients with HF, as it involves coordination across multiple health care settings, various HCPs, and electronic systems. This requires both technological advancements and improved interpersonal strategies to facilitate efficient document sharing and follow-up calls. Improving the Diraya system’s infrastructure, usability, and support tools—along with establishing seminars and an asynchronous chat system—significantly improves the care of patients with HF and adherence to protocols.

Overall, Diraya does not adequately support the TSCP, placing an excessive administrative burden on HCPs. The proposed improvements aim to enhance patient care, reduce hospital readmission rates, and improve overall patient well-being. Achieving these goals will require both technological improvements and the promotion of a collaborative health care culture that strengthens nursing practices and streamlines care transitions between settings.

## Appendix

Multimedia Appendix 1 Flowchart illustration of the visits and decision-making processes within the standardized care pathway for patients with heart failure. Adapted from Comin-Colet et al [16]. CR: cardiac rehabilitation; HC: home care; HD: hospital day; HF: heart failure; HFP: heart failure program; HFpEF: heart failure with preserved Ejection Fraction; HFU: heart failure unit; IM: internal medicine; LVEF: left ventricular ejection; PC: primary care; SCP: standardized care pathway. [PDF file (Adobe Acrobat), 211 kB].

Multimedia Appendix 2 Proposed improvements, key findings, involved key actors, and priority scale based on the thematic analysis and feedback from health care professionals. [PDF file (Adobe Acrobat), 201 kB].

## Abbreviations

AHS: Andalusia Health Service
AI: artificial intelligence
C/IM: cardiologist or internal medicine
CDS: clinical decision support
EHR: electronic health record
HCP: health care professional
HF: heart failure
HN: hospital nurse
MAIC: modelos Asistenciales de Atención al Paciente con Insuficiencia Cardíaca
MDR: medical discharge report
NDR: nursing discharge report
PCN: primary care nurse
PCP: primary care physician
SCP: standardized care pathway
SNHS: Spanish National Health System
STS: sociotechnical system
TSCP: transition standardized care pathway
UX: user experience
WHO: World Health Organization
