# Supplementary figures and images for: Optimizing an Electronic Health Record System Used to Help Health Care Professionals Comply With a Standardized Care Pathway for Heart Failure During the Transition From Hospital To Chronic Care: Qualitative Semistructured Interview Study
Source: JMIR Med Inform. 2025 Apr 15;13:e63665. doi: 10.2196/63665 (PMC12041825; doi:10.2196/63665)

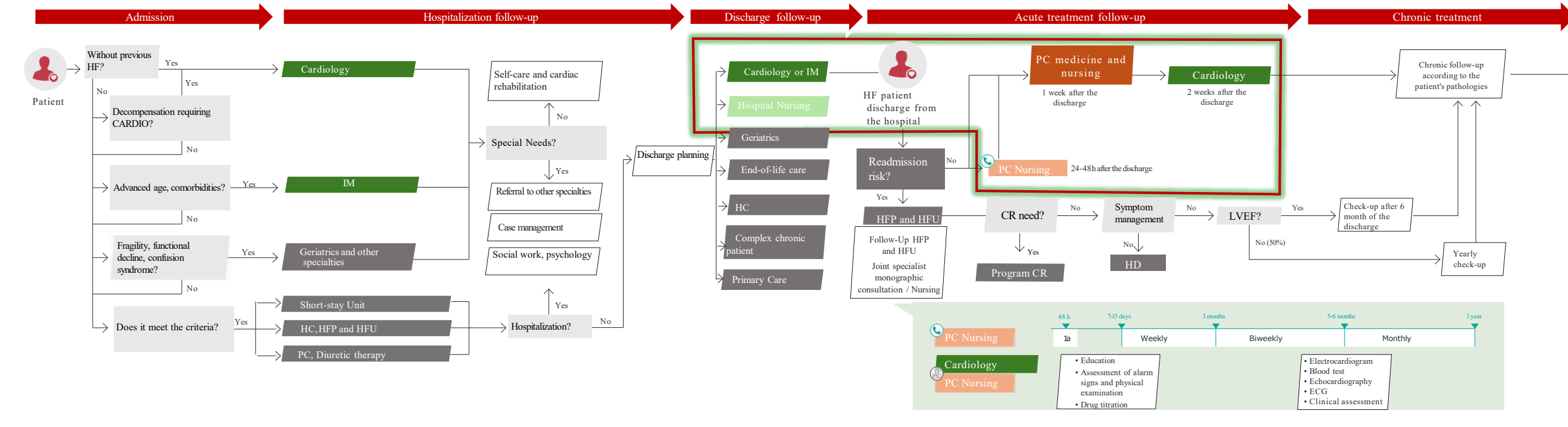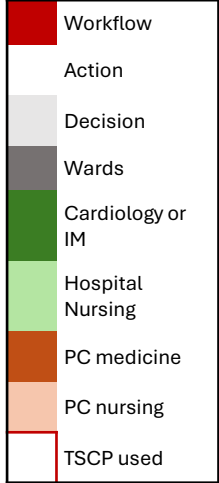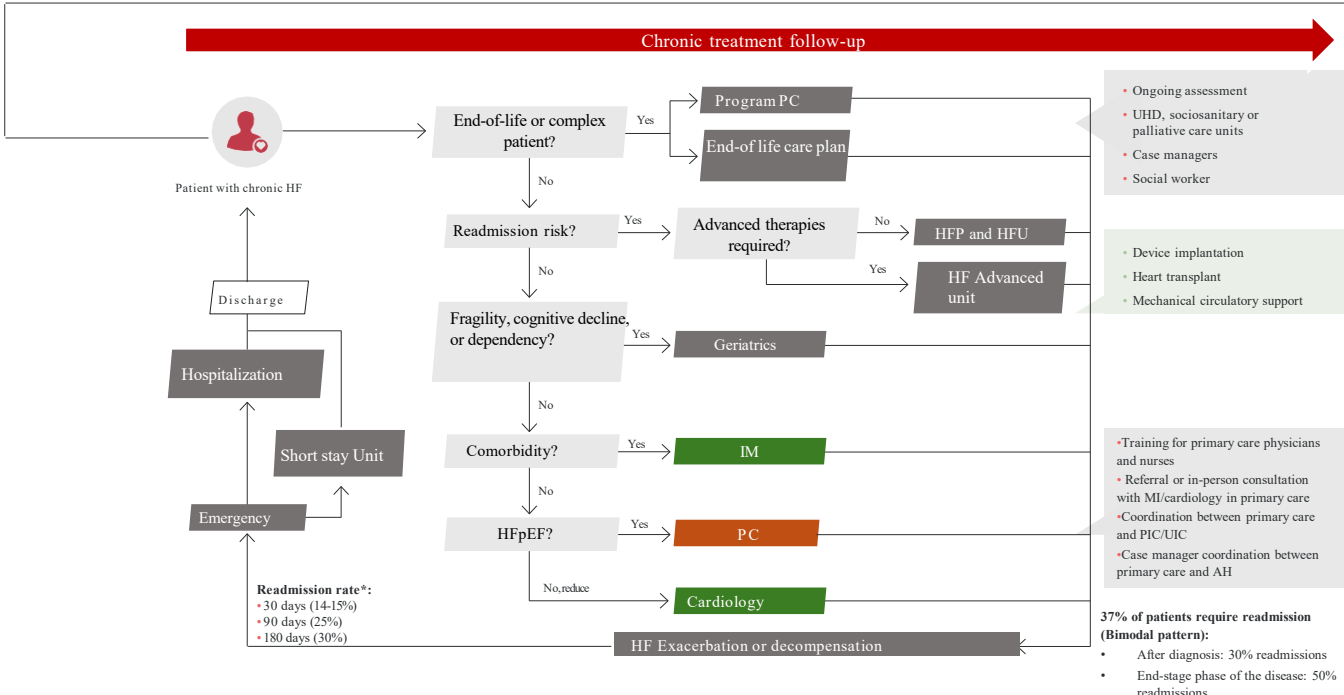

Supplement: Multimedia Appendix 1 [file medinform_v13i1e63665_app1.pdf]
