# Supplementary material for: Optimizing an Electronic Health Record System Used to Help Health Care Professionals Comply With a Standardized Care Pathway for Heart Failure During the Transition From Hospital To Chronic Care: Qualitative Semistructured Interview Study
Source: JMIR Med Inform. 2025 Apr 15;13:e63665. doi: 10.2196/63665 (PMC12041825; doi:10.2196/63665)
Supplement: Multimedia Appendix 2 [file medinform_v13i1e63665_app2.pdf]

Multimedia Appendix 2

Table. Proposed improvements, suggested actions, key findings, involved key actors, and priority scale based on the thematic analysis and feedback from HCPs[a].

Proposed improvements	Suggested actions	Key findings	HCPs	Priority
Creation of a *Care Process* at *Diraya* for HF[b]	• Joint workflow between PCP[c] and PCN[d].<br>• Guidelines for HF patient care.<br>• Management of the patient medical history structure.<br>• Involve both PCN and PCP in the process.<br>• Visits based on the SCP[e].	• Theme 1:<br>○ Clinical report is variable and needs standardization.<br>○ Report manager has low engagement and lacks relevance. Medical history updates depend on physician's workload.<br>• Theme 7:<br>○ Mostly informal (WhatsApp, sticky notes, and direct conversations).<br>○ Shared schedules for PCPs and PCNs exist but are rarely used.	PCP and PCN	Priority 3

		○ Doctor-to-doctor communication between settings is more structured than nurse-to-nurse communication.<br>○ Nurses lack interconsultation options in Diraya.<br>○ PCPs and PCNs receive notifications for patient with HF discharges but lack comprehensive details.		
Consensus on a report manager for hospital and primary care settings	• Establish consensus on common objectives.<br>• Conduct medical assessments during follow up visits.<br>• Define the typology of the visit.<br>• Identify necessary scales.<br>• Remove redundant questionnaire.	• Theme 1:<br>○ Clinical report is variable and needs standardization.<br>○ Report manager has low engagement and lacks relevance.<br>○ Medical history updates depend	PCP, HN[f], and C/IM[g] specialists	Priority 3

	• Incorporate recommendations based on the results.	on physician workload.		
Achieve agreement on the NDR[h]	• Streamline workflows.<br>• Reduce redundant tasks.<br>• Decrease the time HCPs spend navigating between different systems.<br>• Improve the efficiency of data sharing and communication across disciplines.<br>• Improve patient care efficiency and reduce the workload on staff.<br>• Provide specific training to HNs to ensure the knowledge on properly uploading the NDR.	• Theme 2:<br>○ PCNs find the NDR is not personalized and lacks specificity.<br>○ HNs often do not have time to complete the NDR, or if they do, it is too broad.<br>PCPs find the MDR[i] too extensive and time-consuming.<br>• Theme 8:<br>○ Nurses seek a more active role in decision-making during patient transitions.<br>○ Interest in cross-setting collaboration to enhance patient	PCN and HN	Priority 3

		education and continuity of care.<br>○ Diraya training is essential but often unavailable upon job entry due to time constraints.<br>○ Optional training is unlikely to be used unless integrated into work hours.		
Agree with PCPs and C/IMs on essential items for the discharge summary	• Reach a consensus on what information is essential for the summary between primary care and hospital care.<br>• Implement an automated system that does not impose an additional workload on HCPs.	• Theme 2:<br>○ PCNs find the NDR is not personalized and lacks specificity.<br>○ HNs often do not have time to complete the NDR, or if they do, it is too broad.<br>○ PCPs find the MDR too extensive and time-consuming.<br>• Theme 8:	PCP and C/IM specialists	Priority 3

		○ Nurses seek a more active role in decision-making during patient transitions.<br>○ Interest in cross-setting collaboration to enhance patient education and continuity of care.		
CDS[j] tools in medical visits	• Target which new recommendations could be implemented by analyzing the entire medical process.<br>• Influence the decision-making processes of PCP and C/IM during follow up visits.<br>• The system aims to provide real-time decision support that enhances clinical judgments.	• Theme 3:<br>○ Current CDS features are limited to nursing professionals.<br>○ PCNs have concerns about the accuracy and usefulness of the CDS content.<br>○ There is a lack of system-generated recommendations for PCPs.	PCP and C/IM specialists	Priority 2

		○ Patient education on medication and alarm signs is crucial.<br>○ Medication reconciliation can cause confusion, and errors are sometimes overlooked.		
Update nursing CDS	• Update the questionnaire and scales according to guidelines and recommendations for HF practice.	• Theme 3:<br>○ Current CDS features are limited to nursing professionals.<br>○ PCNs have concerns about the accuracy and usefulness of the CDS content.<br>○ There is a lack of system-generated recommendations for PCPs.<br>○ Patient education on medication and alarm signs is crucial.	PCN[d]	Priority 2

		○ Medication reconciliation can cause confusion, and errors are sometimes overlooked.		
Improving the overall UX[k] and usability of the interface	• Iconography<br>• Text size<br>• Personalization<br>• Overall improvement	• Theme 4:<br>○ HCPs become proficient in using the EHR[l] through practice, as it is not intuitive.<br>○ Some professionals found it difficult to learn initially but experience improved usability.<br>○ Font size needs adjustment for readability.<br>○ HNs find the NDR interface cluttered and visually unappealing.<br>○ C/IM professionals	PCP, HN, PCN, and C/IM specialists	Priority 1

		report greater satisfaction with the interface compared to other groups.<br>o PCPs find some icons unclear, particularly for medical history.<br>o Five out of eight participants struggle to interpret icons in the questionnaire.<br>o Hospital professionals rarely consult primary care EHR due to time constraints.<br>o Primary care professionals are more inclined to review hospital EHR but struggle with access.<br>o Accessing hospital EHR is		

		slow and time-consuming.		
Technology infrastructure	• Implement the same technological standards across all AHS[n] facilities involving: new computers, suitable auxiliary monitors, reliable internet connection, and telephones, if needed.	• Theme 5:<br>○ Low quality hardware and software cause delays.<br>○ Frequent internet outages, especially during rain, disrupt workflows.<br>○ Hospital computers often malfunction, affecting documentation efficiency.<br>○ Corporate phone numbers are long and unrecognizable, leading to missed patient follow-ups.<br>○ Some HCPs use personal devices for work-related communication,	PCP, HN, PCN, and C/IM specialists	Priority 3

		raising privacy concerns.		
Interoperability	• Ensure that documents are properly saved in the systems and are accessible through the unified EHR.<br>• For HN, provide training to ensure they know how to use the button, and alternatively, make the button more visible.<br>• Minimize user journeys to access different modules of the EHR.	• Theme 6:<br>○ The Single Health Record system is crucial for patient care transitions but lacks full interoperability.<br>○ PCPs often cannot access complete discharge reports or test results.<br>○ Some reports are not uploaded due to a lack of awareness among hospital staff.<br>○ Accessing hospital EHR from primary care is cumbersome and time-consuming.<br>○ Hospital professionals need complete	PCP, HN, PCN, and C/IM specialists	Priority 2

		primary care information at the time of discharge.		
Scales integration in both settings	• Standardize the scales used throughout the process.<br>• Modify the system to have the same questionnaire data in the 3 modules.<br>• Share and monitor both the scales and patient progress.	• Theme 6:<br>○ Different scales are performed on the patient in both settings, consisting of repetitive assessments used to monitor progress, but the full results are not shared.	PCP. HN, PCN, and C/IM specia lists	Priority 2
Mailbox updates	• Categorize notifications as urgent, important, or not important.<br>• Ensure the handover of tasks to other HCPs if the initial HCP is unavailable.<br>• Include reviewing and updating the mailbox as part of the job objectives.	• Theme 7:<br>○ HNs often experience delays in receiving discharge notifications.<br>○ Informal relationships sometimes fill gaps but are inconsistent.<br>○ PCPs and PCNs receive	PCN and HN	Priority 3

		notifications for patients with HF discharges but lack comprehensive patient details.<br>○ Some patients are overlooked due to unclear alerts.<br>○ The hospital lacks a Diraya mailbox module, breaking communication with primary care.<br>○ The purpose of each patient with HF visit in Diraya is often unclear.		
Chat system for internal and external communication among HCPs	• Implement a chat system for HCPs to facilitate communication.<br>• Avoid using other (private) tools, such as WhatsApp or written notes.	• Theme 7:<br>○ Mostly informal (WhatsApp, post-it notes, and direct conversations).<br>○ Doctor-to-doctor communication between settings	PCP. HN, PCN, and C/IM specialists	Priority 2

	• Standardize communication within the TSCP.	is more structured than nurse-to-nurse communication.		
Organize meetings and trainings for external communication between settings	• Promote meetings and seminars in areas with all involved HCPs<br>• Conduct training sessions to standardize HF practice.	• Theme 7:<br>○ Clinical sessions for knowledge exchange are valuable but require time and resources.<br>• Theme 8:<br>○ Nurses seek a more active role in decision-making during patient transitions.<br>○ Interest in cross-setting collaboration to enhance patient education and continuity of care.<br>○ Diraya training is essential but often unavailable upon job entry	PCP, HN, PCN, and C/IM specialists	Priority 2

		due to time constraints.<br>○ Optional training is unlikely to be used unless integrated into work hours.		
Increase decision-making authority for HN and PCN	• Increase the nurse's involvement in managing patient care within the SCP.<br>• Promote training sessions for nursing staff.<br>• Conduct seminars between HN and PCN.	• Theme 8:<br>○ Nurses seek a more active role in decision-making during patient transitions.<br>○ Interest in cross-setting collaboration to enhance patient education and continuity of care.<br>○ Diraya training is essential but often unavailable upon job entry due to time constraints.<br>○ Optional training is unlikely to be used unless	PCN and HN	Priority 3

		integrated into work hours.		
Diraya traineeship	• Implement mandatory training on *Diraya* for all new employees.<br>• Include this training as part of the standard training curriculum.<br>• Offer additional, nonmandatory training sessions to cover system improvements and updates.	• Theme 8:<br>○ Diraya training is essential but often unavailable upon job entry due to time constraints.<br>○ Optional training is unlikely to be used unless integrated into work hours.	PCP, HN, PCN, and C/IM specialists	Priority 2

[a]HCP: health care professional.

[b]HF: heart failure.

[c]PCP: primary care physician.

[d]PCN: primary care nurse.

[e]SCP: standardized care pathway.

[f]HN: hospital nurse.

[g]C/IM: cardiologist or internal medicine.

[h]NDR: nursing discharge report.

[i]MDR: medical discharge report.

[j]CDS: clinical decision support.

[k]UX: user experience.

[l]EHR: electronic health record.

[m]TSCP: transition standardized care pathway.

[n]AHS: Andalusia health service.
